# Decreased response inhibition in middle-aged male patients with type 2 diabetes

**DOI:** 10.1186/1751-0759-4-1

**Published:** 2010-02-11

**Authors:** Kaya T Ishizawa, Hiroaki Kumano, Atsushi Sato, Hiroshi Sakura, Yasuhiko Iwamoto

**Affiliations:** 1 the Diabetes Center, Tokyo Women's Medical University, 8-1 Kawadacho, Shinjuku-ku, Tokyo 162-8666, Japan; 2 Faculty of Human Sciences, Waseda University, 2-579-15 Mikashima, Tokorozawa-shi, Saitama 359-1192, Japan; 3 Faculty of Human Development, University of Toyama, 3190 Gofuku, Toyama-shi, Toyama 930-8555, Japan

## Abstract

**Background:**

This study was performed to examine whether patients with type 2 diabetes have cognitive deficits associated with the prefrontal cortex (PFC).

**Methods:**

Twenty-seven middle-aged patients with newly diagnosed type 2 diabetes and 27 healthy controls underwent physical measurements and neuropsychological tasks. Response inhibition, reward prediction, and executive function were assessed by the Go/NoGo task, the reversal and extinction tasks, and the Wisconsin Card Sorting Test (WCST). To examine the interactions of being overweight with diabetes on cognitive performance, performance data were analysed by two-way ANCOVA with diabetes and overweight as factors and age as a covariate.

**Results:**

Patients with type 2 diabetes showed significantly decreased response inhibition in the Go/NoGo task (discriminability index: *P *= 0.001). There was an interaction of being overweight with diabetes on reaction time in the Go trials of the Go/NoGo task (*P *= 0.009). Being overweight was related to retained responses to the presentiment of reward in the extinction task (*P *= 0.029). The four groups showed normal cognitive performance in the WCST.

**Conclusions:**

Our results showed that middle-aged, newly diagnosed and medication-free patients with type 2 diabetes have a particular neuropsychological deficit in inhibitory control of impulsive response, which is an independent effect of diabetes apart from being overweight.

## Background

Patients with type 2 diabetes are required to make strict daily decisions for optimal glycemic control; for example, they should resist the temptation of high-fat, high-calorie diets and maintain daily exercise. Although lifestyle improvement is necessary to avoid future complications of diabetes [1], it is often difficult for people to change their daily behaviors.

Previous studies indicated that the ventral PFC regulates human decision making by predicting future rewards and punishments [2,3] and inhibits impulsive thoughts and responses with a distributed cortical network [4], whereas the dorsolateral PFC regulates general cognitive functions such as executive function [5]. Executive function plays a key role in organization of information, carrying out plans, judgment according to outcome and cognitive shifting. On the other hand, many real-world situations elicit actions in which immediate reward is a priority prior to delayed outcome estimated by executive function. For example, overeating in response to environmental cues immediately produces innate pleasure and reduces psychological distress, despite an awareness of future adverse physical outcome. In such conditions, rapid reward prediction or impulsive response to environmental stimuli prevails over the preparations by executive function [2]. While there is accumulating evidence suggesting poorer cognitive performance in executive function in patients with type 2 diabetes compared to those without this disease [6-10], to our knowledge, there has been only one report that patients with type 2 diabetes had cognitive difficulty in response inhibition [10]. This study used a comprehensive neuropsychological test and found selective cognitive deterioration in response inhibition. However, their patients with type 2 diabetes were older adults (mean age = 68.59, range = 55 to 81 years) with an average disease duration of 8.29 years, 65.87% of whom were controlled by oral medication and/or insulin. Therefore, the results regarding cognitive performance in these patients with type 2 diabetes may have been affected by advanced age, disease duration [6,11] and medication [12,13].

Recently, overweight and obese non-diabetic subjects were reported to demonstrate excessive reward prediction [14] and difficulties in inhibition of impulsive responses [15]. Obesity is an important cofactor in type 2 diabetes because the presence of obesity and diabetes together is associated with more severe insulin and leptin resistance than either condition alone. Insulin and leptin are important central signals to regulate PFC functions [16], and these central actions are attenuated in both obesity and diabetes [16,17]. These findings suggest that weight gain and type 2 diabetes may be related to interactive deterioration in cognitive function associated with PFC. In previous studies of type 2 diabetes [6-10], overweight or obese diabetic subjects were not compared with normal-weight diabetic subjects, which made it difficult to determine the independent effects of type 2 diabetes and being overweight and to examine the interaction of being overweight with type 2 diabetes on cognitive function. In this study, we recruited middle-aged, newly diagnosed and medication-free Japanese patients with type 2 diabetes. The average BMI of Japanese diabetic patients is normal compared with the non-diabetic Japanese population [18]. We examined cognitive performance in four groups (overweight patients with type 2 diabetes, normal-weight patients with type 2 diabetes, overweight controls and normal-weight controls), which allowed us to assess cognitive performance using two-way ANOVA with diabetes and overweight as factors.

The aim of our study was to investigate the specific PFC functions including response inhibition and reward prediction in type 2 diabetes. Although it has been suggested that the ventral PFC regulates momentary decision making and behavioural adjustment according to various environmental stimuli with appropriate response inhibition and reward prediction [2-4], the precise effects of type 2 diabetes on such specific PFC functions have not been fully examined. We used particular neuropsychological tasks measuring response inhibition and reward prediction for assessing the ventral PFC functions, and used the Wisconsin Card Sorting Test (WCST) which measures executive function closely related to the dorsolateral PFC functions. This study was performed to test the following hypotheses: 1) Middle-aged patients with type 2 diabetes have cognitive deficits associated with the PFC, including response inhibition and reward prediction. 2) There is an interactive effect of being overweight with type 2 diabetes on these cognitive performances.

## Methods

### Participants

Twenty-seven patients with newly diagnosed type 2 diabetes and 27 healthy control subjects participated in this study. Only male subjects were recruited to avoid the sex differences in cognitive assessment, because sex-related differences have been reported in several cognitive processes, such as response inhibition [19], emotional processing and reward expectation [20], and working memory [21]. To avoid the influence of age, medication, and vascular complications on cognition, we recruited only newly diagnosed middle-aged patients without diabetic complications who had never received any medication for diabetes or the regular and/or intensive program of lifestyle modification at the outpatient clinic of the Diabetes Center of Tokyo Women's Medical University (TWMU) Hospital from July 2005 to June 2007. All patients were diagnosed with type 2 diabetes according to the WHO definition [22]. All patients underwent ophthalmologic assessment on study participation, and those with retinopathy were excluded because diabetic retinopathy is a marker of brain microvascular damage deteriorating PFC function [23]. Educational year-matched healthy control subjects were recruited from among the acquaintances of hospital staff. Inclusion criteria for all participants were as follows: age 30-59 years, right-handed, Japanese speaker and functionally independent. Exclusion criteria for all participants were a history of psychiatric or neurological disorder, dementia, epilepsy, head trauma, loss of consciousness, alcohol and/or substance abuse, micro- or macro-vascular complications, including stroke or cardiovascular disease, and history of regular blood pressure-lowering treatment such as antihypertensive drugs.

To evaluate the effects of type 2 diabetes and being overweight as independent variables, cognitive functions were assessed in four groups: overweight patients with type 2 diabetes, normal-weight patients with type 2 diabetes, overweight controls and normal-weight controls. Height and body weight were measured and body mass index (BMI) was calculated for each subject. According to BMI (kg/m^2^), subjects were categorised as normal weight (18.5-24.9), overweight (25.0-29.9) or obese (≥ 30). Overweight and obese subjects were analysed as a single group because BMI ≥ 25 kg/m^2 ^is defined as obesity in Japan based on data from body-fat percentage. The study was performed in accordance with the Declaration of Helsinki and approved by the Medical Ethics Committee of Tokyo Women's Medical University. All subjects gave their informed written consent to participation in the study.

### Neuropsychological assessment

All subjects underwent neuropsychological examination covering specific cognitive functions. To avoid the influence of medical treatment, patients with type 2 diabetes underwent the examination before commencement of treatment. A trained neuropsychological assessor administered four tests, encouraging subjects for maximum performance, which took 60 min. The test measures were for response inhibition, reward prediction, and executive function.

#### The Go/NoGo task

Inhibitory control of impulsivity was assessed by the Go/NoGo task [4]. The Go stimulus consisting of the letter "N" (2/3 of all trials) and the NoGo stimulus consisting of the letter "H" (1/3) were presented in the center of a computer display in a random order for 100 ms with an interstimulus interval of 2000 ms. The subjects were instructed to press a button with the right index finger in response to the Go stimuli as accurately and quickly as possible, but to withhold their response if the NoGo stimuli was presented. Errors were calculated as the numbers of unplanned responses to the NoGo stimuli (commission errors) and of failed responses to the Go stimuli (omission errors). According to Signal Detection Theory (SDT) [24], there are two kinds of errors and those of correct responses in the Go/NoGo task: 'false alarms' (incorrect 'yes' responses = commission errors), 'misses' (saying 'no' when it should be 'yes'= omission errors), 'hits' (correct response to the Go stimuli) and 'correct rejection' (correct 'no' response to the NoGo stimuli). These performance rates may vary with the task difficulty and the subject's response bias, so that we calculated the z-scores of the hit and false alarm rates by fitting those rates to the cumulative probabilities of the normal distribution. We then calculated discriminability index (d') as: d' = z (hit rate)-z (false alarm rate) to assess response inhibition [24]. Response time was recorded for every response when pressing the button, and the reaction time for responses to the Go stimuli (RT) was used as a variable because it reflects the ability of conflict monitoring [25]. Post-error slowing, which is the reaction time on subsequent trials after unplanned or failed responses, was also calculated because it provides a measure of performance adjustment with error-related feedback [26].

#### The reversal and extinction tasks

Ability to make advantageous actions by predicting future rewards was assessed by the reversal and extinction tasks [3]. In the reversal task, two simple stimuli (circle or star) randomly replaced each other on a computer display, and the subjects gained points for either responding with the correct stimulus or for not responding with the wrong stimulus. One stimulus remained on the display for seven seconds if there was no response, but it disappeared immediately when a response was made. Correct or incorrect was indicated by a pleasant chime (reward) or an unpleasant buzzer (punishment). After reaching nine correct responses, the relationship between the stimuli and the consequences was reversed without advance notice. The extinction was similar except that it became incorrect to respond to either stimulus after reaching nine correct responses. The point could only be gained by refraining from responding, while the errors were calculated as the number of non-refrained responses in the extinction condition.

#### The Wisconsin Card Sorting Test (WCST)

To assess general cognitive functions involved in executive functions, the Japanese version of the Wisconsin Card Sorting Test (WCST) was administered [27], in which four stimulus cards on the upper row and one response card on the lower row were shown on a computer display; the cards had geometric designs divided into three categories: colour, form and number of sets. The subjects were asked to decide how to categorise a response card to the upper four cards, and to search for the correct categorisation by trial-and-error. The achievement scores were related to working memory. Perseverative errors of Milner type were related to cognitive shifting ability, as the errors were caused by adhering to a former category after the classification category had changed.

The four tests were administered in a fixed order: the reversal task, the Go/NoGo task, the WCST and the extinction task. The extinction task was run after the reversal task, with a gap of at least five minutes between the tasks.

### Biomedical and emotional assessment

Blood samples were collected in the fasting state. HbA_1c_, plasma glucose and plasma insulin were measured by latex agglutination immunoassay (Fujirebio, Tokyo, Japan), enzymatic assay with GluDH (Kanto Kagaku, Tokyo, Japan) and radioimmunoassay using a double-antibody/PEG technique (Eiken Kagaku, Tokyo, Japan), respectively. The homeostasis model assessment of insulin resistance (HOMA-IR) was calculated using plasma glucose and insulin. Leptin was measured by radioimmunoassay (Linco Method; Linco, St. Charles, MO).

Clinical depression was assessed by the major depressive episode of the Mini International Neuropsychiatric Interview (M.I.N.I.), Japanese version 5.0.0 [28] for excluding subjects with major depressive disorders and bipolar disorders from this study.

### Statistical analyses

Demographic and biomedical data were analysed by one-way ANOVA, and differences among groups were analyzed using Turkey-Kramer multiple comparison test. *P *value < 0.05 was considered significant.

Neuropsychological performance data were analysed by two-way ANOVA with type 2 diabetes and being overweight as between-subject factors. As age may affect cognitive performance [29], the data were analysed by covariate analysis of variance (ANCOVA) with age as a covariate. One set of data in each of the extinction task and the Go/NoGo task and two sets of data in the WCST were not obtained due to time constraints or technical problems. A main effect was used to determine an independent effect of type 2 diabetes or being overweight on each performance. When both type 2 diabetes and being overweight showed main effects on the same task measure without an interaction, it was considered to show the additive effect of type 2 diabetes and being overweight. An interaction between the two factors was used to evaluate the synergistic (or cancellation) effect of type 2 diabetes and being overweight. If an interaction was found, performance data were analysed by one-way ANCOVA with age as a covariate in each group of diabetes and being overweight. The distribution was adjusted by type III sums of squares in a revision model. When considering multiple ANCOVAs, this analysis was based on a conservative P value (*P *< 0.007) calculated by the Bonferroni method with seven neuropsychological measures, including d', RT and post-error slowing of the Go/NoGo task, the errors after reversal and extinction of the reversal and extinction tasks, and achievement scores and perseverative errors of the WCST. For the purpose of comparing with the results of previous studies, we also note the results surviving a threshold of *P *< 0.05.

The additional analyses with hierarchical linear regression were performed to examine whether a neuropsychological deficit, as well as biomedical changes, contributed to glycemic control (HbA_1c_) in diabetic patients. HbA_1C _was the dependent variable. At step 1, we forced the entry of the neuropsychological measures that showed significant difference between diabetic patients and controls. At step 2, age, BMI, plasma insulin, HOMA-IR, and plasma leptin were entered and selected by the stepwise method with *P *< 0.05 for entry and retention. Statistical significance was set at *P *< 0.05 for these additional analyses.

All data were analysed using SPSS version 12.0 statistical software (SPSS, Chicago, IL).

## Results

### Characteristics

The subjects' characteristics are summarised in Table 1. Twenty-seven subjects were of normal-weight and 27 were overweight or obese. Mean age of the subjects was 41.3 years (range 30-59), with a mean of 16.3 years of education (range 12-20). Age was significantly different between normal-weight patients with type 2 diabetes and control subjects (age; normal-weight patients vs. overweight controls, *P *= 0.001, normal-weight patients vs. normal-weight controls, *P *< 0.001). Years of education were not significantly different between groups. Patients with type 2 diabetes showed higher systolic blood pressure (systolic blood pressure; overweight patients vs. normal-weight controls, *P *< 0.001, normal-weight patients vs. normal-weight controls, *P *= 0.009). Overweight patients with type 2 diabetes showed apparent insulin resistance (HOMA-IR; overweight patients vs. normal-weight controls, *P *= 0.027), and higher plasma leptin concentrations (leptin; overweight patients vs. normal-weight patients, *P *= 0.025, overweight patients vs. normal-weight controls, *P *= 0.021).

**Table 1:** Subject characteristics

	OW patients	NW patients	OW controls	NW controls
N	16	11	11	16
Age (years)	42.9 ± 8.4	50.2 ± 7.1^a, b^	37.0 ± 5.4^a^	36.6 ± 7.9^b^
Range	30-59	38-58	31-48	30-56
Education (years)	15.9 ± 1.2	16.4 ± 1.3	16.6 ± 1.1	16.8 ± 1.3
BMI (kg/m^2^)	29.8 ± 4.5^c, d^	23.4 ± 1.3^a, c^	27.6 ± 3.8^a, e^	21.7 ± 1.4^d, e^
Systolic blood pressure (mmHg)	131.2 ± 9.9^d^	129.6 ± 17.8^b^	122.7 ± 11.2	114.2 ± 8.9^b, d^
Diastolic blood pressure (mmHg)	80.6 ± 11.3	78.6 ± 8.8	82.9 ± 13.2	74.8 ± 12.2
HbA_1C _(%)	8.2 ± 1.6^d, f^	7.6 ± 1.8^a, b^	4.9 ± 0.2^a, f^	4.8 ± 0.3^b, d^
Plasma glucose (mmol/L)	8.4 ± 2.2^d, f^	7.6 ± 1.6^a, b^	5.4 ± 0.3^a, f^	5.3 ± 0.3^b, d^
Plasma insulin (pmol/L)	82.6 ± 70.1	44.4 ± 31.3	54.2 ± 7.6	55.6 ± 11.8
HOMA-IR	4.6 ± 4.4^d^	2.2 ± 1.7	1.9 ± 0.3	1.9 ± 0.4^d^
Plasma leptin (ng/ml)	7.14 ± 4.92^c, d^	2.91 ± 1.47^c^	5.41 ± 3.90	3.48 ± 1.55^d^

Data are given as means ± SD. ^a, b, c, d, e, f^There are significant differences between the groups with the same letters by Turkey-Kramer multiple comparison tests (*P *< 0.05). OW, overweight; NW, normal weight.

### Neuropsychological performance

Table 2 shows both neuropsychological performance data in the four groups and the results of ANCOVA with type 2 diabetes and being overweight as between-subject factors, and age as a covariate. No significant correlation was observed between the results of different neuropsychological tasks.

**Table 2:** Neuropsychological performance of patients and control subjects

	OW patients	NW patients	OW controls	NW controls	DM*	OW*	Interaction*
<Go/NoGo task>							
Discriminability (d')	2.66 ± 0.48	2.39 ± 1.0	3.14 ± 0.59	3.28 ± 0.46	0.001^‡^	0.648	0.215
Commission errors	9.8 ± 3.8	10.1 ± 3.8	6.6 ± 4.2	5.5 ± 2.9	0.002^‡^	0.74	0.463
Omission errors	0.4 ± 0.9	3.8 ± 8.0	0.2 ± 0.4	0.1 ± 0.3	0.049^†^	0.081	0.072
RT (ms)	356 ± 28	330 ± 59	339 ± 37	363 ± 35	0.104	0.629	0.009^†^
Post-error slowing (ms)	379 ± 66	322 ± 68	348 ± 57	343 ± 40	0.525	0.035^†^	0.054
<Reversal and extinction tasks>							
Errors after reversal	1.7 ± 3.0	1.2 ± 0.4	1.1 ± 0.3	1.0 ± 0.4	0.977	0.358	0.448
Errors after extinction	7.6 ± 3.7	6.4 ± 4.0	8.7 ± 5.2	5.1 ± 3.0	0.894	0.029^†^	0.340
<WCST>							
Achievement scores	5.4 ± 1.0	4.6 ± 1.3	5.9 ± 0.6	5.6 ± 0.5	0.218	0.117	0.773
% perseverative errors	2.7 ± 6.4	8.2 ± 10.1	4.2 ± 9.0	3.2 ± 5.3	0.458	0.296	0.147

Neuropsychological data are given as means ± SD. *Data were analysed by covariate analysis of variance (ANCOVA) with age as a covariate, and the main effects and interaction of type 2 diabetes and being overweight are shown as *P *values. ^†^*P*<0.05, ^‡^*P *< 0.007 with ANCOVA. OW, overweight; NW, normal weight; DM, type 2 diabetes mellitus; RT, reaction time in unplanned responses to the Go stimuli.

Type 2 diabetes showed a significant main effect on d' in the Go/NoGo task (F(1,49) = 12.585, *P *= 0.001). Patients with type 2 diabetes showed significantly decreased response inhibition: d' = 2.55 ± 0.14 and 3.22 ± 0.10 (means ± SE) in patients and control subjects, respectively. Additional analyses were performed to investigate which errors of commission and omission were more dominant for decreased response inhibition. In the error made, type 2 diabetes showed a more apparent main effect on commission errors (F(1,49) = 10.83, *P *= 0.002) than on omission errors (F(1,49) = 4.063, *P *= 0.049). RT did not differ significantly between groups. Overweight subjects showed longer post-error slowing than normal-weight subjects: post-error slowing = 367.7 ± 12.7 ms and 335.1 ± 10.2 ms (means ± SE) in overweight and normal-weight subjects, respectively (F(1,49) = 4.74, *P *= 0.035). RT and post-error slowing showed significant correlation with d' in patients with type 2 diabetes (RT; Pearson *r *= 0.582, *P *= 0.002, post-error slowing; Pearson *r *= 0.556, *P *= 0.003). However, the adjustment with RT or post-error slowing did not change the results of two-way ANCOVA on d' in the Go/NoGo task (data not shown). As patients with type 2 diabetes showed higher systolic blood pressure than normal-weight controls and higher blood pressure has been shown to affect cognitive performance [10], the results of diabetes-related group differences were further controlled by ANCOVA with systolic blood pressure as a covariate. The adjustments with systolic blood pressure did not change the results of diabetes-related group differences in d', commission or omission errors in the Go/NoGo task (data not shown).

There was a significant interaction of type 2 diabetes with being overweight on RT (F(1,49) = 7.516, *P *= 0.009). We thus performed stratified ANCOVAs with age as a covariate as follows. To examine the effect of type 2 diabetes on RT, each of normal-weight and overweight subjects was separately analyzed with one-way ANCOVA with diabetes as a between-subject factor and with age as a covariate. Next, each of diabetic and non-diabetic subjects was separately analyzed with one-way ANCOVA with being overweight as a between-subject factor and with age as a covariate to examine the effect of being overweight on RT. Type 2 diabetes showed a trend for an effect on shorter RT for normal-weight subjects (*P *= 0.062), but not for overweight subjects (*P *= 0.553). Being overweight showed an effect on longer RT for diabetic patients (*P *= 0.033), but not for non-diabetic subjects (*P *= 0.094), suggesting that normal-weight diabetic patients had worse performance in RT than overweight diabetic patients. There was also a trend for an interaction on post-error slowing (F(1,49) = 3.909, *P *= 0.054). Type 2 diabetes showed no effects on post-error slowing for both normal-weight and overweight subjects (data not shown). Being overweight showed an effect on longer post-error slowing for diabetic patients (*P *= 0.025), but not for non-diabetic subjects (*P *= 0.872), suggesting similar results to RT.

In the reversal and extinction tasks, type 2 diabetes did not show main effects on mean errors. Being overweight did not show a significant main effect on the mean error after reversal. However, overweight subjects showed continuous responding for the previously reward stimulus in the extinction task, and the mean errors after extinction were significantly different between overweight and normal-weight subjects: the mean error after extinction was 8.1 ± 0.8 and 5.6 ± 0.7 (means ± SE) in overweight and normal-weight subjects, respectively (F(1,49) = 5.095, *P *= 0.029).

In the WCST, the mean achievement scores of the four groups were within normal limits (≥ 4) [27]. Neither type 2 diabetes nor being overweight showed main effects or interactions on the achievement scores and perseverative errors of the WCST.

### Neuropsychological deficits and glycemic control

Patients with type 2 diabetes showed significantly decreased response inhibition (d') in the Go/NoGo task. We then analyzed the effect of d' on HbA_1c _with hierarchical linear regression to examine if this neuropsychological deficit contributed to glycemic control in patients with type 2 diabetes. At step 1, we forced the entry of d'. At step 2, age, BMI, plasma insulin, HOMA-IR, and plasma leptin were entered and selected by the stepwise method with *P *< 0.05 for entry and retention. The d' as well as BMI explained a significant proportion of the variance of HbA_1c _(*R*^2 ^= 0.287, *P *= 0.024, d': *β *= -0.433, *P *= 0.029, BMI: *β *= 0.433, *P *= 0.029). Age, plasma insulin, HOMA-IR, and plasma leptin were excluded from the model. As patients with type 2 diabetes showed higher systolic blood pressure, we assessed whether blood pressure would change the result of d' on HbA_1c_. Systolic blood pressure added to the former model did not change the result of d', and systolic blood pressure was excluded from the model (data not shown).

## Discussion

The main finding of this study was that inhibitory control of impulsive responses was decreased in newly diagnosed middle-aged patients with type 2 diabetes. The Go/NoGo task is commonly used to assess the ability to inhibit impulsive responses, as poor performance in this task presents as rapid, unplanned reactions to environmental stimuli without regard to negative consequences of these reactions [4,30]. The Go/NoGo task has good test-retest reliability [31] and provides several indices of response inhibition, such as the error made and the reaction times (RT and post-error slowing) [25,26]. In this study, we calculated discriminability index (d') from hits and false alarm rates to estimate the task difficulty considering the subject's response bias. As expected, d' was significantly decreased in patients with type 2 diabetes. Further, we found that decreased d' was significantly associated with increased HbA_1C _in diabetic patients, suggesting the lack of inhibitory control may be related to the aggravation of type 2 diabetes. Additional analysis indicated a greater difference in the number of commission errors than in omission errors between diabetic patients and controls in the Go/NoGo task. The increase in commission errors suggests failure of impulsivity control despite both knowledge of adverse consequences and a desire to quit [4,30,31]. Several studies have indicated a significant correlation between self-reported impulsivity and increased commission errors in the Go/NoGo task [32,33]. In patients with diabetes, temptation to eat is frequently cued by specific people (e.g., a friend always carrying sweets), place (e.g., a particular shop) and events (e.g., parties). Appropriate behavior modification thus depends on the patient's ability to inhibit impulsive thoughts and actions cued by these environmental stimuli. As this was a cross-sectional study, we cannot make any definitive conclusions regarding a causal relationship. However, our study included only newly diagnosed patients with type 2 diabetes, suggesting the possibility that the neuropsychological deficits in response inhibition may contribute to the behavioral problems leading to chronic lifestyle-related diseases, such as type 2 diabetes. It is also possible that the metabolic changes with diabetes affect brain functions and cause neuropsychological deficits [7,8]. Several studies indicated that metabolic improvement resulted in improved cognitive function in people with type 2 diabetes [9,34]. Further longitudinal studies will be useful to detect progression or improvement of neuropsychological deficits associated with metabolic changes.

A Previous study [10] demonstrated that diabetes-related difference was apparent in the selective cognitive domain requiring a contribution of speed and response inhibition in older patients with type 2 diabetes. They demonstrated a significant decrease in response inhibition across the two late-life age groups in young-old and old-old subjects (mean age and range: young-old, 63.64, 55-69 years old; old-old, 75.59, 71-80 years old), using the Hayling test and Color Trails 2 task. We confirmed their findings with regard to the abnormality of response inhibition using the Go/NoGo task in younger patients (mean age and range: 45.89, 30-59 years old) than in their study. We included only patients with newly diagnosed type 2 diabetes according to the WHO definition and with no diabetic complications. Therefore, our patient characteristics reduced the influence of cognitive decline associated with age, disease duration-related brain damage [6,11] and the effects of medication [12,13] in type 2 diabetes. It is important to note that both Yeung *et al. *[10] and the present study found similar relations between diabetes and response inhibition abnormality in different patient populations, which strongly supports the suggestion that the deficit in the cognitive domain of response inhibition may be a particular manifestation of diabetes-associated cognitive dysfunction that is robust across different age groups.

With regard to the contribution of speed to response inhibition, the possible influence of the subject's motor performance, error monitoring and immediate adjustment after committing errors should be addressed [25,30]. With regard to motor performance, Hershey *et al. *demonstrated decreased d' in the Go/NoGo task in Parkinson disease patients with improved motor performance due to subthalamic nucleus stimulation [35], suggesting that response inhibition cannot be explained easily by changes in motor performance. As previous studies of type 2 diabetes indicated no impairments in simple motor function assessed by the simple reaction time [7,10] or Trail Making Test A [8], it is unlikely that simple motor function made the main contribution to the present results. Next, the results of RT and post-error slowing reflect the ability of conflict monitoring and performance adjustment with error-related feedback in the Go/NoGo task [25,26]. Consistent with the previous findings that longer RT and post-error slowing is related to better performance adjustment with appropriate error monitoring [25,36], RT and post-error slowing was positively related to better performance (increased d') in patients with type 2 diabetes in the present study. However, the results of group comparison of d' did not change after adjustment with RT or post-error slowing, suggesting that the decreased response inhibition in diabetic patients may relate to the different neurological function from error monitoring and adjustments. We found significant decrease in d' but not in RT and post-error slowing in patients with type 2 diabetes. Kaiser *et al. *also reported decreased d' but similar RT in patients with major depression, with the findings of reduced event-related potential in the frontal lobe in patients with decreased d', suggesting that specific impairment of impulsivity control is associated with PFC dysfunction in depressive patients [37]. RT and post-error slowing were reported to reflect anterior cingulate cortex (ACC) function [25,26,36]. A previous study using functional magnetic resonance imaging demonstrated dissociated roles of ACC and PFC in response inhibition, such that ACC function is engaged when an ongoing action needs to be quickly changed and PFC function is more important in changing attitudes toward future consequences [38]. These findings support the possibility that the inhibitory failure observed in diabetic patients could be mainly explained by cognitive impairment of impulsivity control associated with PFC function rather than by deficits in motor performance, error monitoring and adjustment.

The results of RT and post-error slowing suggested that there may be interactive effects of type 2 diabetes with being overweight on reaction times in the Go/NoGo task. The direction of interactions on RT and post-error slowing was different from our prediction. RT and post-error slowing were shorter in normal-weight patients than in overweight patients with type 2 diabetes, whereas these reaction times were not different between normal-weight and overweight non-diabetic control subjects. We assumed that one of the major differences between normal-weight and overweight patients with type 2 diabetes was the ability of insulin secretion [18]. Normal-weight patients with type 2 diabetes showed low plasma insulin concentration, whereas overweight patients with type 2 diabetes showed apparent insulin resistance and high plasma insulin concentration. Previous studies suggested that insulin resistance is a determinant of free fatty acids which are important in tryptophan metabolism and brain serotonin concentrations, and that overweight subjects who are insulin resistant may have higher brain serotonin concentration [39,40]. On the other hand, normal-weight diabetic patients with insulin deficiency may have reduced brain serotonin activity [41] and may be more impulsive than overweight patients with type 2 diabetes. Brain serotonin concentration level was reported to affect ACC activation during the Go/Nogo task [42], and these findings are consistent with the present results. However, there have been conflicting results with regard to ACC function in type 2 diabetes [43,44]. One study reported attenuated ACC activation [43] and the other did not find differential ACC activation between patients with type 2 diabetes and controls [44]. These previous studies [43,44] did not indicate the BMI of the subjects nor compare overweight and normal-weight patients with type 2 diabetes, which made it difficult to evaluate an interaction of being overweight with type 2 diabetes on ACC function, and this issue should be confirmed in further studies.

We also found that overweight and obese subjects showed significant performance difference in errors after extinction in the extinction task. Overweight and obese subjects retained responses to stimuli showing the presentiment of reward after the extinction condition of the former reward in the extinction task although the result did not survive a conservative *P *value. Overweight and obese subjects had difficulty in refraining from inappropriate responses and their behavior seemed guided by previous experiences of reward, which is consistent with the results of a previous study that indicated decision-making deficits with addictive reward-seeking in obesity [14]. It is increasingly recognized that much of excess caloric intake in obesity is driven by the rewarding properties of palatable food [45]. People learn associations between environmental stimuli (e.g. a box contains palatable food) and the reinforcement value (reward with innate pleasure), and hedonic factors such as taste and the memorized reward may play a large role in continuous food consumption even after energy homeostasis has been fulfilled. Increased errors in the extinction task suggest a failure in breaking previously learned associations between stimuli and reinforcement value [3]. Therefore, overweight and obese subjects may have difficulties in correcting their food consumption when the reinforcement value of the stimuli has changed (e.g. adverse physical outcome by taking palatable food), which will result in a failure of dietary improvement and further weight gain. However, the degree of obesity in our subjects was lower than in several previous studies indicating relation of cognitive decline with BMI [46,47], which may have been responsible for our limited findings. Further studies of larger numbers of obese subjects are needed to investigate this relation.

The present results regarding working memory and executive function were markedly different from those of previous studies that suggested significant impairment of executive function in subjects with type 2 diabetes [6-9] or overweight subjects [46,47]. The mean achievement scores of the WCST were all within normal limits (≥ 4) and the perseverative errors were not different among the groups. Although a previous neuropsychological study indicated that the achievement scores of the WCST were negatively correlated with the number of commission errors in the Go/NoGo task [48], there were no associations of the WCST variables with the results of the Go/NoGo task or the extinction task. Therefore, the poorer performance in these tasks cannot be attributed to reduced executive function. The normal performance in the WCST may have been due to the subjects' age and high educational background in this study, because higher age and lower education level have lowering effects on the WCST [27,49]. A previous study indicated that the WCST score is strongly associated with age-related cortical atrophy and white matter lesions (WMLs) in the PFC [50]. It is possible that newly diagnosed middle-aged patients with no complications do not have marked cognitive decline associated with age or disease duration-related brain damage [6].

This study had the following strengths. First, we assessed cognitive performance in the four groups and analysed performance data using two-way ANOVA with diabetes and overweight as factors, which allowed us to find specific cognitive dysfunction in type 2 diabetes. Second, we studied younger subjects than those included in previous studies [6-10] with exclusion of diabetic vascular complications, which helped reduce the influence of age and vascular cognitive decline in assessing the cognitive functions. Third, we recruited newly diagnosed and medication-free patients, which allowed us to exclude the effects of oral medication [12] and exogenous insulin [13] in assessing the cognitive functions.

There were several limitations in the study. First, the study population included only men because men and women show important differences in clinical conditions in which cognitive deficits are implicated. Several studies have reported sex-related differences in response inhibition [19,51], reward expectation [20], and working memory [21]. However, especially in response inhibition, there have been conflicting reports suggesting no difference in behavioral and brain-functional results in the Go/NoGo task [25]. Although menstruation and pregnancy should be carefully excluded [21], further studies including middle-aged women will provide more information on the cognitive function associated with the Go/NoGo task. Second, the results should be considered carefully for multiple ANCOVAs. Based on a conservative *P *value (*P *< 0.007), only the results of decreased inhibitory control in patients with type 2 diabetes remained significant. Third, the degree of obesity in our subjects was lower than in several previous studies indicating obesity-related cognitive decline [46,47], which may have been responsible for our limited findings. Further studies comparing a more severely obese group and non-obese group with or without diabetes would be helpful to find specific and interactive cognitive deficits between obesity and diabetes. Finally, we cannot definitely conclude there were any causal relationships as this was a cross-sectional study.

## Conclusion

In conclusion, this study revealed a particular neuropsychological deficit, *i.e.*, difficulty in inhibitory control of impulsive responses in middle-aged, newly diagnosed type 2 diabetes with or without being overweight, the clinical significance of which remains to be investigated in future studies.

## Competing interests

The authors declare that they have no competing interests.

## Authors' contributions

KTI collected and analyzed data, and drafted the article. HK conceptualized the study, supported the data analysis, and led the writing of the article. AS supervised the study and assisted with writing the article. HS assisted in planning the study, collecting data, and preparing the article. YI supervised the direction of the study. All authors read and approved the final manuscript.
